# Identification of tri-phosphatase activity in the biogenesis of retroviral microRNAs and RNAP III-generated shRNAs

**DOI:** 10.1093/nar/gku1247

**Published:** 2014-11-26

**Authors:** James M. Burke, Clovis R. Bass, Rodney P. Kincaid, Christopher S. Sullivan

**Affiliations:** The University of Texas at Austin, Institute for Cellular and Molecular Biology, Center for Synthetic and Systems Biology, Center for Infectious Disease and Department of Molecular Biosciences, 1 University Station A5000, Austin TX 78712-0162, USA

## Abstract

Transcripts possessing a 5′-triphosphate are a hallmark of viral transcription and can trigger the host antiviral response. 5′-triphosphates are also found on common host transcripts transcribed by RNA polymerase III (RNAP III), yet how these transcripts remain non-immunostimulatory is incompletely understood. Most microRNAs (miRNAs) are 5′-monophosphorylated as a result of sequential endonucleolytic processing by Drosha and Dicer from longer RNA polymerase II (RNAP II)-transcribed primary transcripts. In contrast, bovine leukemia virus (BLV) expresses subgenomic RNAP III transcripts that give rise to miRNAs independent of Drosha processing. Here, we demonstrate that each BLV pre-miRNA is directly transcribed by RNAP III from individual, compact RNAP III type II genes. Thus, similar to manmade RNAP III-generated short hairpin RNAs (shRNAs), the BLV pre-miRNAs are initially 5′-triphosphorylated. Nonetheless, the derivative 5p miRNAs and shRNA-generated 5p small RNAs (sRNAs) possess a 5′-monophosphate. Our enzymatic characterization and small RNA sequencing data demonstrate that BLV 5p miRNAs are co-terminal with 5′-triphosphorylated miRNA precursors (pre-miRNAs). Thus, these results identify a 5′-tri-phosphatase activity that is involved in the biogenesis of BLV miRNAs and shRNA-generated sRNAs. This work advances our understanding of retroviral miRNA and shRNA biogenesis and may have implications regarding the immunostimulatory capacity of RNAP III transcripts.

## INTRODUCTION

MicroRNAs (miRNAs) are small RNAs (∼22 nt) that regulate eukaryotic gene expression (1,2). Most miRNAs are generated via an established biogenesis pathway. RNA polymerase II (RNAP II) transcribes longer primary miRNA (pri-miRNA) transcripts containing one or multiple stem-loop structures in which miRNAs are embedded. The RNase III enzyme Drosha cleaves these stem-loop structures to liberate precursor miRNA (pre-miRNA) hairpins (3–7). Pre-miRNAs are subsequently exported to the cytosol and processed by Dicer (8–10), yielding ∼22 nt duplex RNAs (3). Typically, one RNA strand is loaded into the RNA induced silencing complex (RISC). RISC-associated miRNAs then guide mRNA association via partial sequence complementary, generally resulting in repression of gene expression (11–13).

Of the approximately 300 viral miRNAs identified to date [reviewed in (14–17)], most are generated through the canonical miRNA biogenesis pathway. However, a subset of viral miRNAs are produced via noncanonical routes. For example, murid gammaherpesvirus 68 (MHV-68) expresses RNAP III-transcribed pri-miRNA transcripts (18–21) that are processed by tRNaseZ to generate pre-miRNAs (22). Herpesvirus saimiri (HVS) expresses RNAP II-transcribed Sm class U RNAs (HSURs) that are processed by the integrator complex to generate pre-miRNAs (23). Subsequently, both the MHV-68 and the HVS pre-miRNAs then enter the canonical biogenesis pathway and are processed by Dicer to produce miRNAs.

Our lab and others have identified miRNAs derived from five hairpin structures that are encoded in an intragenic region of the bovine leukemia virus (BLV) genome (24,25). Previous work demonstrated that BLV-miR-B4 mimics miR-29 (24). As miR-29 overexpression is associated with B-cell neoplasms (26,27), this finding has provided new insight into the mechanism of BLV-associated tumorigenesis. Analysis of the biogenesis of BLV-miR-B4 demonstrated that it is derived from a subgenomic RNAP III transcript independent of Drosha processing (24). This established a general retroviral miRNA biogenesis pathway that permits miRNA expression while avoiding cleavage of the viral mRNA and genomic RNA (24). However, the detailed mechanisms of BLV pre-miRNA expression and generation have remained unresolved.

Here, we characterize the BLV miRNA biogenesis pathway in depth. We demonstrate that each BLV pre-miRNA is directly transcribed by RNAP III from an independent, compact RNAP III gene. Furthermore, though our data show that the BLV pre-miRNAs are predominantly 5′-triphosphorylated, we demonstrate that the derivative BLV 5p miRNAs, as well as 5p small RNAs (sRNAs) derived from manmade RNAP III-generated short hairpin RNAs (shRNAs), harbor a 5′-monophosphate. These results uncover a previously unappreciated tri-phosphatase activity involved in the biogenesis of BLV miRNAs and shRNA-generated sRNAs.

## MATERIALS AND METHODS

### Plasmids

The BL3.1 BLV miRNA cassette was amplified by polymerase chain reaction (PCR) using BLV_cassette primers (Supplementary Table S1) from genomic BL3.1 DNA and cloned into pIDTK xho1/xba1 sites. The individual pBLV miRNA expression vectors were constructed by fill-in PCR from primers encoding the individual BLV pri-miRNAs (NC_001414; Supplementary Table S1) and cloned into the xho1/xba1 sites of pIDTK plasmid. The Rluc BLV 3′ UTR plasmid was constructed by PCR amplification of the BLV Gag-Pol 3′ UTR from pBLV913 plasmid [a gift from L. Mansky, University of Minnesota, Minneapolis (28)] using BLV_3′ UTR primers (Supplementary Table S1), digesting the fragment with sal1/spe1 and cloning the fragment into the pcDNA3.1dsLuc2CP xho1/xba1 sites. To make the Rluc BLV 3′ UTR TM construct, a sequence encoding the BLV cassette with terminator mutations (BLV913_miRNAs_TM_gblock; Supplementary Table S1) was synthesized (Integrated DNA Technologies), amplified and extended using the BLV913_miRNAs_TM primers (Supplementary Table S1), and cloned into the ApaL1 sites of pIDT_BLV913 (pIDT_BLV913_TM). The BLV Gag-Pol 3′ UTR _TM was then amplified from pIDT_BLV913_TM using BLV_3′ UTR primers (Supplementary Table S1), digested with sal1/spe1, and cloned into the pcDNA3.1dsLuc2CP xho1/xba1 sites. The pBLV913_miRNA expression vector was made by PCR amplification from FLK-BLV genomic DNA and cloned into pcDNA3.1 Bgl II/ Apa1 sites. The B1 and B4 A-box, B-box and terminator mutants were made by two-step fill-in PCR using oligos (Supplementary Table S1) encoding the point mutations, extended by using the respective universal_B1_s or unviseral_B4_ primers, and cloned into the xho1/xba1 sites of pIDT. The BLV miRNA RISC reporter constructs were made by fill-in PCR using BLV_RR oligos (Supplementary Table S1), which encode two complimentary sites to an individual BLV miRNA, and cloned into the xho1/xba1 sites of pcDNA3.1dsLuc2CP. shRNA plasmids used were pRNA-U6.1-siLuc (GenScript), psiRNA-hH1nEGFP G2 (InvivoGen) and pSUPER antiCox1 (Oligoengine). All plasmid constructs were sequence verified (ICMB core facilities, UT Austin, USA).

### Cell culture

HEK293T cells were obtained from American Type Culture Collection (ATCC) and maintained in Dulbecco's modified Eagle's medium supplemented with fetal bovine serum (FBS; Cellgro; 10% vol/vol). BL3.1 cells were obtained from ATCC and maintained in RPMI 1640 supplemented with FBS (10% vol/vol).

### Northern blot analysis

Northern blots were performed as described in (29). Briefly, total RNA was extracted from cells using PIG-B (30), fractionated on 15% polyacrylamide gelelectrophoresis (PAGE)-urea gel, transferred to Amersham Hybond –N+ membrane (GE Healthcare) and probed with indicated DNA oligos (Supplementary Table S1).

### RNAP III dependence

HEK293T cells were co-transfected with 500-ng SV40-miR-S1 vector, MGHV-miR-M1-7 vector and 1 μg of BLV miRNA expression vector (either Rluc BLV 3′ UTR or pIDT_BL3.1 miRNAs) using Lipofectamine 2000 (Invitrogen) and then treated 2 h later with a final concentration of 50 μg/ml of α-amanitin (Sigma). Total RNA was extracted 24 h post transfection and northern blot analysis was performed.

### BLV miRNA expression from individual vectors

HEK293T cells (6-well format, 70% confluency) were transfected with 2 µg of pBLV miRNA expression vectors using Turbofect (Thermoscientific) transfection reagent. RNA was extracted 24 h later and northern blot analysis was performed.

### Analysis of BLV B1 and B4 RNAPIII transcriptional elements

HEK293T cells (12-well format, 70% confluency) were co-transfected with 500-ng SV40-miR-S1 and 1.5 µg of each individual B1 or B4 vector using Lipofectamine 2000. Thirty hours post transfection RNA was harvested and northern blot analysis was performed using the B1_3p probe or B4_3p_(s) probe (Supplementary Table S1).

### Semi-quantitative PCR of Rluc mRNA

HEK293T cells were transfected with either 1 µg of Rluc BLV 3′ UTR or Rluc BLV 3′ UTR TM expression vectors using Lipofectamine 2000 (Invitrogen). Total RNA was extracted and 6 µg was used for northern blot analysis. Five micrograms of RNA was treated with DNaseI (NEB) according to manufacturer's instructions. RNA was purified by ammonium acetate precipitation. One microgram of RNA was converted to cDNA using a polyT_20_ primer and superscript III reverse transcriptase (Invitrogen). Control reactions without Superscript reverse transcriptase were performed for each RNA sample. The RT reactions were diluted to 80 µl. One microliter of cDNA was PCR amplified using Taq polymerase (NEB; 94°C 60 s, 94°C 20 s, 55°C 20 s, 72°C 30 s) and BLV_cassette primers or GAPDH primers (Supplementary Table S1). Ten microliters of the PCR reaction was removed at various cycle numbers (gradient) and fractionated on 1.2% agarose TAE gel.

### *In vitro* Dicer assay

To assay BLV pre-miRNAs from cells, RNA was isolated from HEK293T cells transfected with the BLV miRNA expression vectors and fractionated on 15% PAGE-urea gel. Pre-miRNA-sized RNAs (40–120 nt) were then gel-purified as previously described (31). Five-hundred nanograms of RNA was then treated with or without Dicer (Genlantis), according to manufacturer's instructions, for 1 and 3 h. Northern blot analysis was then performed using probes specific for each BLV miRNA (Supplementary Table S1). To assay Dicer kinetics of a purified 5′-triphosphorylated BLV pre-miRNA, we generated a BLV-pre-miR-B1 mimic using the AmpliScribe™ T7-Flash™ transcription kit (Epicentre) and oligos encoding the T7 promoter and B1 pre-miRNA (Supplementary Table S1) according to the manufacturer's instructions. Note that the 5′-Adenosine of BLV-pre-miR-B1 was changed to a 5′-Guanosine due to sequence constraints for T7-mediated transcription. The RNA was extracted with PIG-B and fractionated on 15% PAGE-urea gel. The pre-miRNA band (55 nt) was then gel-excised and purified. The pre-miRNA was flash annealed in 100-mM Tris (pH 7.5), 30-mM NaCl and 3-mM MgCl_2_. A 50-µl Dicer reaction (15-nM pre-miR-B1, 1-mM adenosine triphosphate, 2.5-mM MgCl_2_, 1X Buffer, 0.5 units of recombinant human Dicer) was incubated at 37°C. Five microliters of the reaction was removed and added to the loading buffer/stop solution at the indicated time points. Northern blot analysis using the BLV-miR-B1_3p probe was then performed.

### 5′-end characterization of small RNAs

Two-hundred micrograms of total RNA from HEK293T or HEK293T NoDice(2-20) (32) transfected with shRNA vectors or BLV miRNA expression vectors (pcDNA BLV913 or pBLV miRNA vectors) or BL3.1 cells was fractionated on 15% PAGE-urea. Small RNAs (<70 nt) were isolated via gel excision (one-tenth of tRNA, U6 RNA and 5S RNA were retained), eluted in 1-M NaCl, concentrated using a viva spin column (GE) and recovered by ammonium acetate precipitation. One to five micrograms of RNA was treated with or without 1 µl of RNA 5′ polyphosphatase (Epicentre) in a 20 µl reaction and incubated for 1 h at 37°C. RNA was then purified via ammonium acetate precipitation. Five-hundred nanogram–one microliter of recovered RNA was then treated with or without 1 µl of Terminator^TM^ exonuclease (Epicentre) in a 20-µl reaction and incubated for 3 h at 30°C. The RNA was then subject to northern blot analysis using probes specific for each BLV miRNA (Supplementary Table S1).

### Small RNA sequencing

HEK293T cells (6-well format) were transfected with 2 µg of BLV miRNA expression vectors using Lipofectamine 2000. RNA was extracted 48 h post transfection and fractionated on a 15% PAGE-urea gel. Small RNAs (<70 nt) were isolated as previously described (31) and treated with or without RNA 5′ polyphosphatase. RNA was then recovered via ammonium acetate precipitation. 5′-end characterization as described above, was performed to confirm miRNA expression and dephosphorylation by the RNA 5′ polyphosphatase treatment. Small RNA libraries were then prepared (GSAF, UT Austin, USA) for Illumina small RNA sequencing (RNA-seq) using the multiplex small RNA library prep set (New England Bio Labs) and sequenced on a Illumina HiSeq 2500. Adapter sequences were trimmed from the reads using custom Python scripts. The preprocessed reads were then mapped to the respective pri-miRNA sequences using the SHRiMP2 software package (33). To analyze the fold change of the 5′-start position of putative B5 pre-miRNAs with RNA 5′ polyphosphatase, we counted RNA-seq reads of RNAs approximately pre-miRNA length (>50 nt), in which the 5′-nucleotide perfectly mapped to the BLV B5 miRNA locus (30 nt upstream and 54 nt downstream of the 5′-end of BLV-miR-B5 5p).

### RISC reporter assays

Five nanograms of Renilla (pcDNA3.1dsRluc) RISC activity reporter vector, 5-ng firefly reporter (pcDNA3.1dsLuc2CP) and 1 μg of individual BLV miRNA expression vectors were co-transfected into HEK293T cells (12-well format) using Lipofectamine 2000. Twenty-four hours after transfection, the cells were harvested and processed with the Dual-Glo Luciferase Assay System (Promega) according to the manufacturer's instructions. The luciferase activity was measured on a Luminoskan Ascent luminometer (Thermo Electronic). All reporter expression is normalized to the empty Renilla luciferase vector and irrelevant miRNA expression vector [either TTV_ AB038624-miR (34) or MGHV-miR-M1-7 (24)].

### Structural predictions of pri-miRNAs

Secondary structures of pre-miRNAs were generated using the RNA folding forum on the Mfold web server (35).

## RESULTS

### BLV pri-miRNAs are individually transcribed by RNAP III

**Figure 1.**
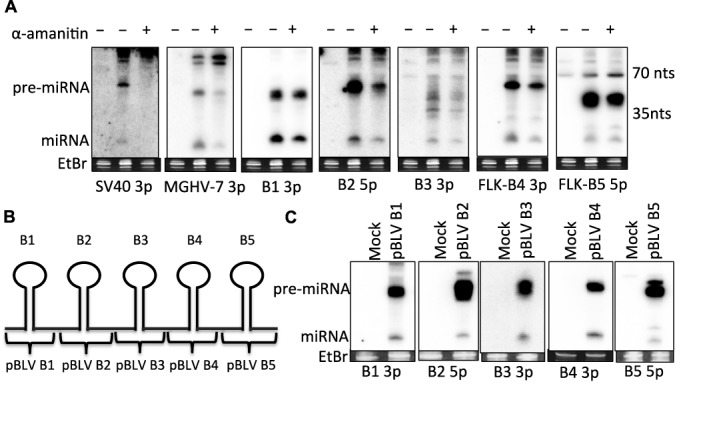
BLV pri-miRNAs are individually expressed via RNAP III. (**A**) Northern blot analysis of HEK293T cells of co-transfected with the control RNAP II-driven SV40-miR-S1 and RNAP III-driven MGHV-miR-M1-7 miRNA expression vectors, and a plasmid encoding the BLV913 miRNA cassette (Rluc BLV 3′ UTR) and then treated with or without 50-nM α-amanitin. (**B**) Schematic diagram illustrating the plasmid constructs encoding each individual BLV (NC_001414) pri-miRNA hairpin. (**C**) Northern blot analysis of HEK293T cells transfected with each individual pBLV miRNA expression vector.

We previously demonstrated that BLV-miR-B4 is transcribed by RNAP III and predicted that the other BLV miRNAs would likewise be transcribed by RNAP III (24). To assay RNAP III-dependence for the other BLV miRNAs, HEK293T cells were co-transfected with the RNAP II control SV40-miR-S1 vector, the RNAP III control MGHV-miR-M1-7 vector and a plasmid encoding the BLV miRNA cassette. The cells were then treated with or without α-amanitin (50 µg/ml), which is sufficient to inhibit RNAP II-mediated transcription. As expected, α-amanitin treatment decreased SV40-miR-S1 expression while MGHV-miR-M1-7 expression was largely unaffected (Figure 1A). Importantly, α-amanitin treatment did not considerably decrease expression of the BLV pre- or mature-miRNAs. This indicates that all the BLV miRNAs are expressed independent of RNAP II-mediated transcription. In agreement with previous work (24,25), these data support that all the BLV miRNAs are transcribed by RNAP III.

Because the BLV pre-miRNA hairpins are clustered in the same region of the genome, we wanted to determine whether each individual BLV pri-miRNA region is sufficient to give rise to each individual pre-miRNA, or if their expression/processing depends on elements flanking the miRNA loci. To assay this, we cloned each BLV pre-miRNA region (∼30 bp upstream and downstream of the pre-miRNA hairpin) into a vector that lacks a known mammalian promoter (Figure 1B). HEK293T cells were transfected with the pBLV miRNA expression vectors. Northern blot analysis showed that each individual pBLV expression vector expressed the pre- and mature-miRNAs (Figure 1C). These results demonstrate that all the BLV pri-miRNAs can be individually transcribed by RNAP III and processed to yield pre-miRNAs. Thus, sequence elements in the regions flanking the immediate pre-miRNA genomic regions are not required for individual BLV pre-miRNA expression.

### BLV pri-miRNAs are expressed from independent, compact RNAP III genes

**Figure 2.**
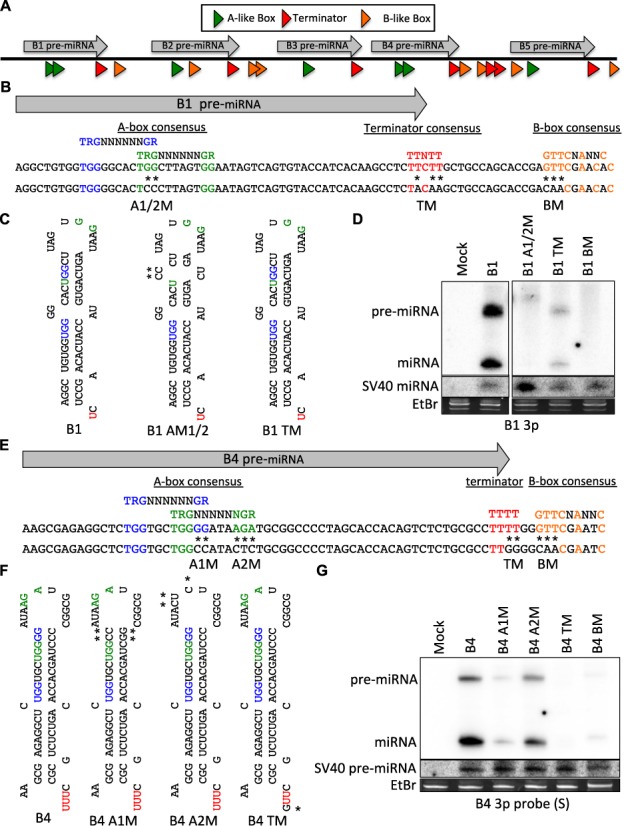
BLV pri-miRNAs are expressed from independent, compact RNAP III genes. (**A**) Schematic diagram (not to scale) illustrating the RNAP III transcriptional elements within the BLV miRNA-encoding region. Each transcriptional element is denoted as an arrow and color-coded: A boxes (green), terminators (red) and B boxes (orange). (**B**) Illustration of the position/sequence of the transcriptional elements within the BLV-miR-B1-encoding region (NC_001414). The consensus sequence for each transcriptional element is colored: A box-1 (blue), the A box-2 (green), terminator (red) and B box (orange). Mutations are indicated by (*) and the name of the mutation is indicated below (e.g. A1M denotes the construct in which the A box-1 consensus sequence is mutated as indicated). (**C**) Predicted pre-miRNA secondary structure of the B1 variants. The (*) indicate point mutations. (**D**) Northern blot analysis of HEK293T cells co-transfected with the SV40-miR-S1 miRNA expression vector and each individual BLV-miR-B1-variant expression vector. (**E**) Illustration of the position/sequence of the transcriptional elements within the BLV-miR-B4-encoding region (NC_001414). The consensus sequence for each transcriptional element is colored: A box-1 (blue), the A box-2 (green), terminator (red) and B-box (orange). Mutations are indicated by (*) and the name of the mutation is indicated below. (**F**) Predicted secondary structure of the BLV-miR-B4 variants. The (*) indicates a point mutation relative to B4. (**G**) Northern blot analysis of HEK293T cells co-transfected with the SV40-miR-S1 miRNA expression vector and each individual BLV-miR-B4-variant expression vector.

The above data suggest that each BLV pre-miRNA is individually expressed by RNAP III. Three general classes of RNAP III transcription initiation are differentiated by the composition and arrangement of various promoter elements [reviewed in (36)]. Analysis of the BLV miRNA-encoding region revealed that each BLV pre-miRNA region contains characteristics of RNAP III type II-initiated RNAs (24). RNAP III type II initiated RNAs, such as tRNAs, contain two intragenic promoter elements, the A box (TGRNNNNNNGR) and the B box (GTTCNANNC), which bind transcription factors that then assemble RNAP III at the transcription start site (36). Most BLV miRNA loci contain one or two A-like box sequences in the 5p arm/loop of the pre-miRNA ∼30–60 bp upstream of a predicted B-like box sequence (Figure 2A). The only exception to this is BLV-miR-B3, which was not predicted by our analysis to encode a B-like box immediately downstream of the predicted B3 pre-miRNA hairpin. Unlike typical RNAP III type II genes, the B box sequences are likely not intragenic, as the predicted terminator sequences (polyT_4–6_) are positioned between the A box and the B box at the 3′-end of each encoded pre-miRNA hairpin.

To determine whether the predicted RNAP III transcriptional elements within the immediate proximity of a BLV pre-miRNA locus promote miRNA expression, we generated BLV-miR-B1 variants in which the consensus sequence of the predicted A boxes (B1 A1/2M), the terminator (B1 TM) or the B box (B1 BM) was mutated (Figure 2B and C). HEK293T cells were co-transfected with the control SV40-miR-S1 vector and each pBLV-miR-B1 expression construct. Northern blot analysis revealed that mutation of the predicted A boxes and B box markedly decreased B1 pre-miRNA and mature miRNA expression (Figure 2D), suggesting that these sequence elements promote B1 expression. Interestingly, mutation of only A box-1 did not reduce the overall B1 expression but resulted in a longer pre-miRNA by ∼5 nt, which appeared to decrease Dicer processing efficiency (Supplementary Figure S1A–C). Conversely, mutation of A box-2 decreased overall B1 expression (Supplementary Figure S1A–C). These results indicate that A box-2 is required for promoting RNAP III transcription initiation whereas A box-1 may influence the RNAP III transcription start site and/or pri-miRNA processing. Mutation of the putative terminator sequence reduced B1 pre-miRNA and miRNA production (Figure 2D). This indicates that RNAP III transcription termination at the 3′-end of the B1 hairpin is required for B1 pri-miRNA expression. These data show that multiple *cis* elements are required for appropriate BLV-miR-B1 transcription and processing.

To demonstrate that the B1 miRNA gene architecture is conserved in another BLV miRNA, we performed similar mutagenesis studies on BLV-miR-B4 (Figure 2E and F). Mutation of the consensus sequences of the predicted A box-1 and the B box significantly decreased pre- and mature-miRNA production, indicating these sequence elements promote RNAP III transcription initiation of pri-BLV-miR-B4. Mutation of the predicted A box-2 slightly decreased B4 expression (Figure 2G), suggesting that it may enhance transcription initiation of pri-BLV-miR-B4. Mutation of the terminator sequence at the 3′-end of the B4 hairpin also abolished B4 expression, indicating that RNAP III transcription termination at the 3′-end of the B4 hairpin is required for B4 pre-miRNA production. Combined, these data demonstrate that each BLV pri-miRNA is expressed from an individual, compact RNAP III type II gene.

### RNAP II transcripts do not efficiently generate BLV miRNAs

**Figure 3.**
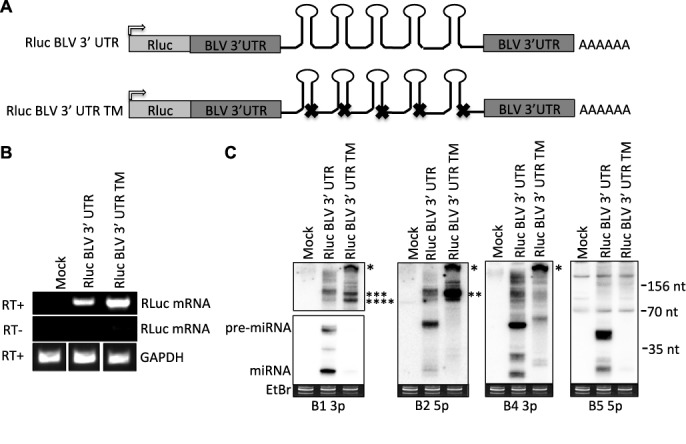
mRNA transcripts encoding the BLV pri-miRNAs do not generate miRNAs. (**A**) Schematic diagram of the Rluc BLV 3′ UTR construct in which the BLV Gag-Pol 3′ UTR encompassing the miRNA cassette was cloned into *Renilla* luciferase expression vector. The terminator sequence at the 3′ end of each BLV pri-miRNA hairpin was mutated (X) to generate the Rluc BLV 3’UTR TM construct. Gray arrow indicates CMV promoter. (**B**) Semi-quantitative PCR amplification of the BLV miRNA-encoding region was performed from HEK293T cells transfected with either the Rluc BLV 3′ UTR or Rluc BLV 3′ UTR TM expression vectors to confirm expression the Rluc mRNA encoding the BLV pri-miRNAs. (**C**) Northern blot analysis from HEK293T cells transfected with either the Rluc BLV 3’ UTR or Rluc BLV 3’UTR TM expression vectors. Asterisks indicate putative RNAP III transcriptional read-through transcripts (*400 nt; **110 nt; ***100 nt; ****80 nt).

Our previous work demonstrated that Drosha does not process RNAP II transcripts that contain the BLV pre-miRNA region (24). However, whether the miRNAs could be efficiently generated from longer RNAP II or RNAP III transcripts via a Drosha-independent mechanism, similar to the MHV-68 (22) or HSUR (23) miRNAs, remained unclear. To address this, we cloned the BLV Gag-Pol 3′ UTR, which encompasses the miRNA cassette, into the 3′ UTR region of the RNAP II-driven *Renilla* luciferase vector (Rluc BLV 3′ UTR). We then generated minimal point mutations in the terminator sequences at the 3′-end of all the pre-miRNA hairpins (Rluc BLV 3′ UTR TM) (Figure 3A). Our above data predict that these mutations will abolish RNAP III transcription termination, while structural prediction suggests that this mutant transcript will maintain the gross structures of the pri-miRNA hairpins (data not shown). HEK293T cells were transfected with either the Rluc BLV 3′ UTR or Rluc BLV 3′ UTR TM expression vectors. Semi-quantitative PCR confirmed that the Rluc BLV 3′ UTR TM mRNA (containing the BLV miRNA region but lacking the RNAP III termination signals) was expressed at comparable levels to the mRNA transcript containing the wild-type BLV 3′ UTR (Figure 3B). However, northern blot analysis revealed that the Rluc BLV 3′ UTR TM construct produced considerably less BLV pre- and mature-miRNAs (Figure 3C). Slower migrating RNAs, consistent with the size of predicted RNAP III read-through transcripts, were abundantly produced by the Rluc BLV 3′ UTR TM construct but not the Rluc BLV 3′ UTR construct. This shows that longer RNAP III read-through transcripts encoding multiple BLV pri-miRNAs do not efficiently generate pre-miRNAs. Combined with our previous work (24), these data demonstrate that neither Drosha-dependent nor Drosha-independent mechanisms efficiently process longer transcripts containing the BLV pre-miRNA region to generate BLV pre- or mature-miRNAs.

### BLV pre-miRNAs are RNAP III primary transcripts

**Figure 4.**
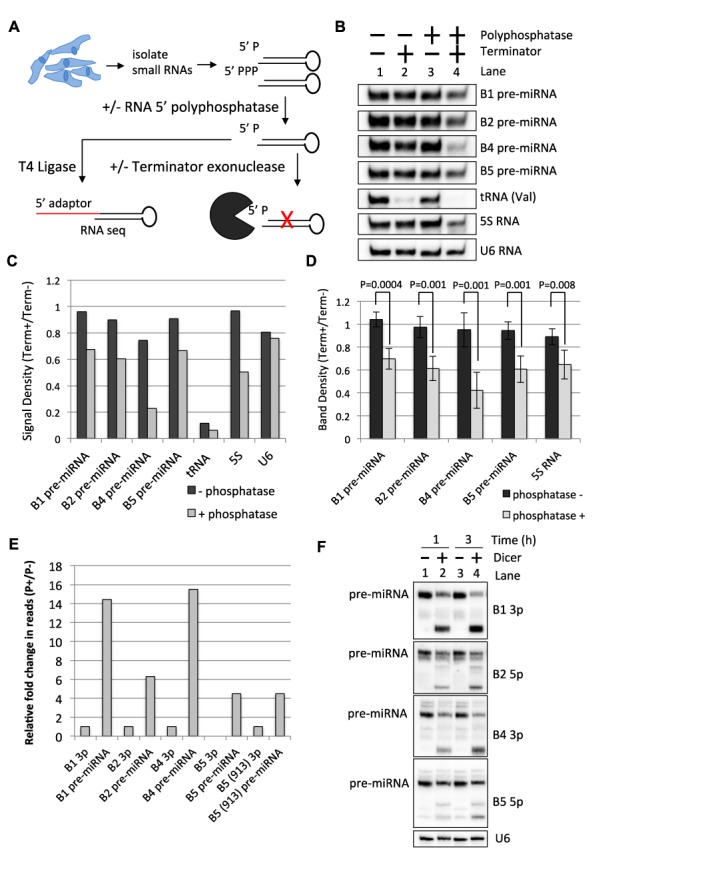
BLV pre-miRNAs are RNAP III primary transcripts. (**A**) Schematic diagram of the 5′-end characterization of BLV pre-miRNAs. (**B**) Northern blot analysis on small RNAs purified from HEK293T cells transfected with the individual BLV miRNAs, treated with or without RNA 5′ polyphosphatase, and subsequently treated with or without Terminator^TM^, a 5′-monophosphate-dependent exonuclease. (**C**) Graphical representation of the band densities ratios (Terminator^TM^+/Terminator^TM^−) from (B) with or without RNA 5′ polyphosphatase treatment. (**D**) Graphical representation of the band density ratio (Terminator^TM^+/Terminator^TM^−) of the BLV pre-miRNAs and the 5S RNA with or without RNA 5′ polyphosphatase treatment as determined by northern blot analysis from four independent biological replicates. The bars represent the average ratio +/− the standard deviation (*n* = 4). (**E**) Fold change relative to the respective 3p miRNAs in the number of small RNA-seq reads of RNAs consistent with pre-cursors to the BLV miRNAs with or without RNA 5′ polyphosphatase. (**F**) Northern blot analysis of BLV pre-miRNAs isolated from HEK293T cells transfected with BLV miRNA expression vectors and treated with or without Dicer *in vitro*.

Our above data (Figures 2 and 3) demonstrated that RNAP III transcription termination generates the 3′-end of the pre-miRNAs. We next wanted to determine how the 5′-end of the BLV pre-miRNAs is generated. The A box promoter elements of type II initiated RNAP III RNAs are located ∼8–20 nt downstream of the transcription initiation site [reviewed in (37)]. As the A box promoter elements necessary for transcription are ∼10–20 nt downstream of the predicted 5′-end of the BLV pre-miRNAs (Figure 2), this suggests that RNAP III transcription initiation may directly determine the 5′-end of the BLV pre-miRNAs. However, longer RNAs spanning the pre-miRNA that contain additional nucleotides on the 5′-end are sometimes observed upon transient transfection of BLV miRNA expression vectors (24) (Supplementary Figure S2A). Therefore, we also considered the possibility that the 5′-end of the BLV pre-miRNAs might derive from nuclease-mediated cleavage of longer ‘pri-cursor’ transcripts. To determine whether the 5′-end of the BLV pre-miRNAs is generated via nuclease-mediated cleavage from longer ‘pri-cursor’ RNAs or directly via RNAP III transcription initiation, we analyzed the 5′-end chemistry of the pre-miRNAs. While primary RNAP III transcripts intrinsically contain a 5′-triphosphate, nuclease-cleaved pre-miRNAs would be expected to contain a 5′-monophosphate or a 5′-hydroxyl. We transfected HEK293T cells with BLV miRNA expression vectors, extracted total RNA and then size-fractionated to enrich for small RNAs (<150 nt). The small RNA preparation was then incubated *in vitro* with or without Terminator^TM^, a 5′-monphosphate-dependent exonuclease (Figure 4A). As expected, northern blot analysis revealed that 5′-monophosphorylated mature tRNA (Val) decreased with Terminator^TM^ treatment, while the 5′-triphosphorylated 5S RNA was unaffected (Figure 4B, Lanes 1 and 2, C; Supplementary Figure S2B). Importantly, the BLV pre-miRNAs were largely unaffected by Terminator^TM^ treatment. This suggests that a large fraction of the BLV pre-miRNAs lacks a 5′-monophosphate.

To test whether the BLV pre-miRNAs contain a 5′-triphosphate, we pre-treated the small RNA preparation with RNA 5′ polyphosphatase, which removes the γ and β phosphates from 5′-triphosphorylated RNA, and then incubated with Terminator^TM^. Under these conditions, Terminator^TM^ treatment now markedly decreased the 5S RNA and BLV pre-miRNA signals (30–75%). In contrast, U6 RNA, which is O-methylated on the 5′-gamma phosphate (38) and presumably not a substrate for the RNA 5′ polyphosphatase, remained unaffected by Terminator^TM^ treatment (Figure 4B, Lanes 3 and 4, C; Supplementary Figure S2B). We note that Terminator^TM^ treatment did not completely degrade the BLV pre-miRNAs under these conditions, which we attribute is likely due to inhibitory structure and/or incomplete Terminator^TM^ activity. Nonetheless, multiple biological replicates of this assay demonstrated that the ratio (Terminator^TM^+/Terminator^TM^−) of the 5S RNA and the BLV pre-miRNAs is significantly decreased with RNA 5′ polyphosphatase pre-treatment (Figure 4D). Importantly, the MGHV-miR-M1-7 pre-miRNA, which is structurally similar to the BLV pre-miRNAs but is expected to be 5′-monophosphorylated (22), was also only partially susceptible (∼50%) to Terminator^TM^ (Supplementary Figure S2C). These results strongly suggest that a large fraction of the BLV pre-miRNAs contain a 5′-triphosphate.

As an independent assay to determine the 5’ end chemistry of the BLV pre-miRNAs, we treated our small RNA fractions with or without the RNA 5′ polyphosphatase and then performed small RNA-seq. To generate our small RNA libraries, we utilized T4 RNA ligase to ligate the 5′ adaptors to the 5′-end of the small RNAs. Because T4 RNA ligase catalyzes the ligation of 5′-monophosphorylated RNAs to RNAs with a 3′-hydroxyl group, RNAs with a 5′-triphosphate are not readily included in the library unless the 5′-triphosphate is first converted to a 5′-monophosphate (39). Accordingly, RNA 5′ polyphosphatase treatment did not appreciably increase the relative read count for miR-92a (an abundant host miRNA), while the 5′ read counts for the 5S RNA increased ∼1.8-fold (Supplementary Figure S2D). RNA 5′ polyphosphatase treatment increased the read counts of likely pre-miRNAs relative to the respective BLV 3p miRNAs (we note that pre-miRNA-sized RNAs mapping to the B3 locus were not observed under these assay conditions) (Figure 4E; Supplementary Table S2). Combined, these results suggest that the RNAP III transcription start site defines the 5′-end of each BLV pre-miRNA.

We next wanted to ensure that the bands we observe migrating at the typical pre-miRNA size (50–55 nt), as observed via northern blot analysis (Figure 4B), are indeed capable of being the Dicer substrates that give rise to the BLV miRNAs. We gel-purified pre-miRNA-sized RNAs (∼40–120 nt) from HEK293T cells transfected with the BLV miRNA expression vectors and incubated the RNA *in vitro* with purified Dicer. Northern blot analysis demonstrated that the pre-miRNA-sized RNAs were indeed processed by Dicer into RNAs that correspond to the size of the respective BLV miRNAs (Figure 4F; Supplementary Figure S2E). As the BLV pre-miRNAs are largely 5′-triphosphorylated (Figure 4B–E), this suggests that Dicer may directly processes the 5′-triphosphorylated pre-miRNAs. To more directly assay this, we used a substrate that is ∼100% 5′-triphosphorylated (*in vitro* transcribed BLV-pre-miR-B1 mimic) and performed an *in vitro* Dicer kinetics assay. We observed that the 5′-triphosphorylated BLV-pre-miR-B1 mimic was readily processed by Dicer into sRNAs of ∼22 nt in length (Supplementary Figure S2F). These data demonstrate that Dicer is able to directly process 5′-triphosphorylated BLV pre-miRNAs.

### The BLV 5p miRNAs contain a 5′-monophosphate

**Figure 5.**
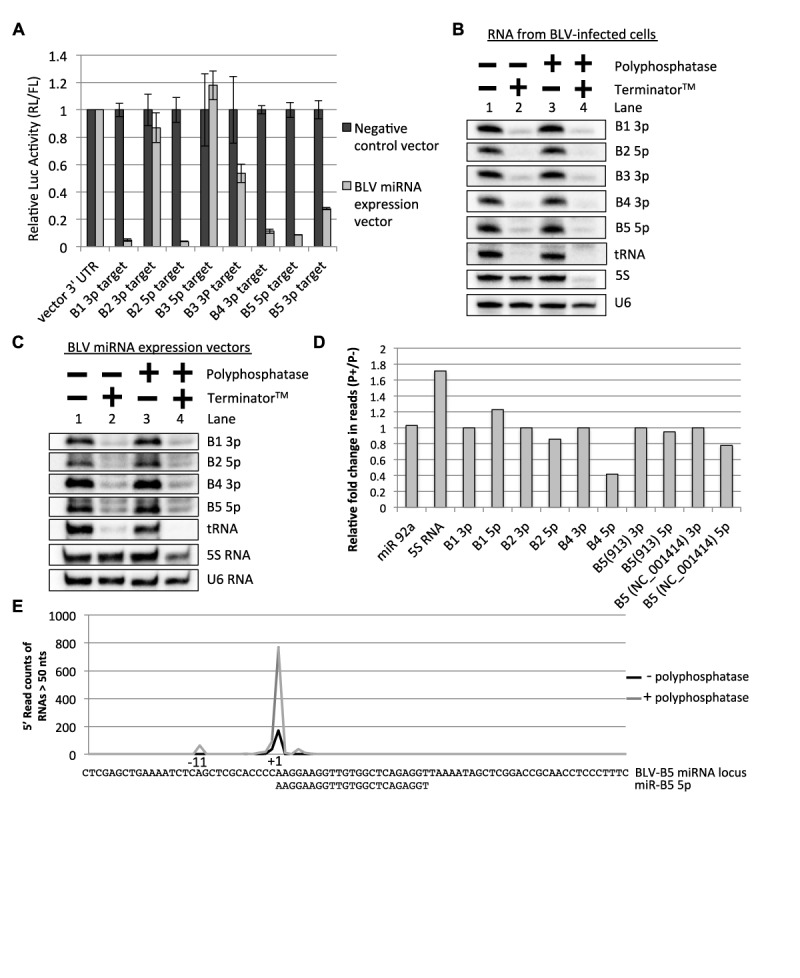
BLV 5p miRNAs contain a 5′-monophosphate. (**A**) RISC reporter assay. HEK293T cells were co-transfected with either irrelevant miRNA expression vector or an individual BLV miRNA expression vector and the respective Rluc vector encoding two complementary target sites to the indicated miRNAs. The graph depicts the average luciferase activity ratio (Ren/FF) +/− standard deviation (*n* = 3) normalized to vector 3′ UTR. (**B**) 5′-end characterization of BLV miRNAs from BLV-infected cells. Small RNAs were isolated from BL3.1 cells, treated with the indicated enzymes and northern blot analysis was performed. (**C**) 5′-end characterization of BLV miRNAs. Northern blot analysis was performed on small RNAs purified from HEK293T cells transfected with BLV miRNA expression vectors and treated with the indicated enzymes. (**D**) Graph representation of the fold change in the number of small RNA-seq reads, relative to the respective predominant 3p miRNA isoform, for each predominant BLV 5p miRNA isoform with or without RNA 5′ polyphosphatase pre-treatment. (**E**) Graphical representation of the 5′-read counts of pre-miRNA-sized RNAs (>50 nt in length) that map to the BLV (NC_001414) B5 miRNA locus (30 nt upstream and 54 nt downstream of the 5′-end of the predominant BLV-miR-B5 5p isoform) with and without RNA 5′ polyphosphatase pre-treatment. The number of reads of the pre-miRNA-sized RNAs was normalized to the predominant BLV-miR-B5 3p miRNA isoform in each dataset (Supplementary Table S2). The nucleotide positions are relative to the 5′-end of the predominant BLV-B5 5p miRNA isoform (+1).

The above data suggest that a considerable fraction of the BLV pre-miRNAs contain a 5′-triphosphate. Thus, we reasoned that the derivative 5p miRNAs might also be 5′-triphosphorylated, and if so, may interfere with their association with RISC. To address whether the abundant 5p miRNAs associate with and are active in RISC, we generated individual RISC activity reporter constructs for both BLV 5p and 3p miRNAs. Two perfectly complementary sequences to each BLV miRNA were inserted into the 3′ UTR region of *Renilla* luciferase. HEK293T cells were then co-transfected with each *Renilla* luciferase RISC reporter plasmid and relevant miRNA expression vector. We observed that both 5p and 3p reporter expression decreased significantly when co-transfected with relevant BLV miRNA expression vectors (Figure 5A). These results demonstrate that BLV 5p miRNAs are active in RISC.

Because BLV 5p miRNAs are active within RISC, this argues that either 5′-triphosphorylated sRNAs can associate with RISC or that these 5p miRNAs are 5′-monophosphorylated, similar to endogenous host miRNAs. Re-examination of our published RNA-seq data (24) suggested that at least a fraction of the 5p miRNAs are 5′-monophosphorylated, as the 5p miRNAs derived from BLV-miR-B2 and BLV-miR-B5 are readily sequenced via small RNA-seq from BL3.1 cells (a bovine B-cell line with proviral BLV genome). To determine the proportion of BLV 5p miRNAs containing a 5′-monophosphate, we isolated small RNAs from BL3.1 cells and performed 5′-end characterization as described above. As expected, Terminator^TM^ treatment degraded 5′-monophosphorylated RNAs: BLV-miR-B1 3p, BLV-miR-B3 3p, BLV-miR-B4 3p and tRNA, while the 5′-triphsophorylated 5S RNA was unaffected (Figure 5B, Lanes 1 and 2). Terminator^TM^ treatment also markedly decreased BLV-miR-B2 5p and BLV-miR-B5 5p without RNA 5′ polyphosphatase pre-treatment. This demonstrates that the majority of BLV-miR-B2 5p and BLV-miR-B5 5p miRNAs contain a 5′-monophosphate. Similar results were obtained from HEK293T cells transfected with individual BLV miRNA expression vectors (Figure 5C). As an independent assay, we treated our small RNA preparation from HEK293T cells transfected with the BLV miRNA expression vectors with or without RNA 5′ polyphosphatase and then performed small RNA-seq analysis. Unlike what we observed for the BLV pre-miRNAs (Figure 4E), RNA 5′ polyphosphatase treatment did not substantially increase the read counts for BLV-miR-B2 5p or BLV-miR-B5 5p relative to the respective 3p miRNAs (Figure 5D). Combined, these data argue that a considerable fraction of BLV-miR-B2 5p and BLV-miR-B5 5p are 5′-monophosphorylated.

To gain insight into the underlying mechanism that gives rise to the 5′-monophosphaylated BLV 5p miRNAs, we plotted the 5′-read counts of pre-miRNA-sized RNAs (>50 nt) in our small RNA-seq data that map to the BLV B5 miRNA locus (NC_001414). As demonstrated in our above data (Figure 4E), the 5′-read counts of the likely B5 pre-miRNA considerably increased (∼4.5 fold) with RNA 5′ polyphosphatase treatment relative to BLV-miR-B5 3p (Figure 5E; Supplementary Table S2). Importantly, the 5′-end of the predominant isoform of BLV-B5-miR 5p (Figure 5E), which does not increase with RNA 5′ polyphosphatase treatment (Figure 5B-D; Supplementary Table S2), is co-terminal with the 5′-end of this likely B5 pre-miRNA. Thus, the 5′-nucleotide identity is conserved between the RNAP III-transcribed B5 pre-miRNA and its derivative 5′-monophosphorylated 5p miRNA. We obtained similar results for other BLV miRNAs (Supplementary Table S2). Further analysis also revealed that many of the 5p miRNA isoforms contain a 5′-purine consistent with a pyrimidine/purine (−1/+1) junction in the template DNA—a characteristic of RNAP III initiation sites (40–42) (Figure 5E; Supplementary Table S2). Combined, these data argue that a tri-phosphatase activity converts the 5′-end of the BLV pre-miRNAs and/or pre-miRNA derivatives into a monophosphate.

### Conversion of the BLV pre-miRNAs to a 5′-monophosphate does not precede Dicer processing in cells

**Figure 6.**
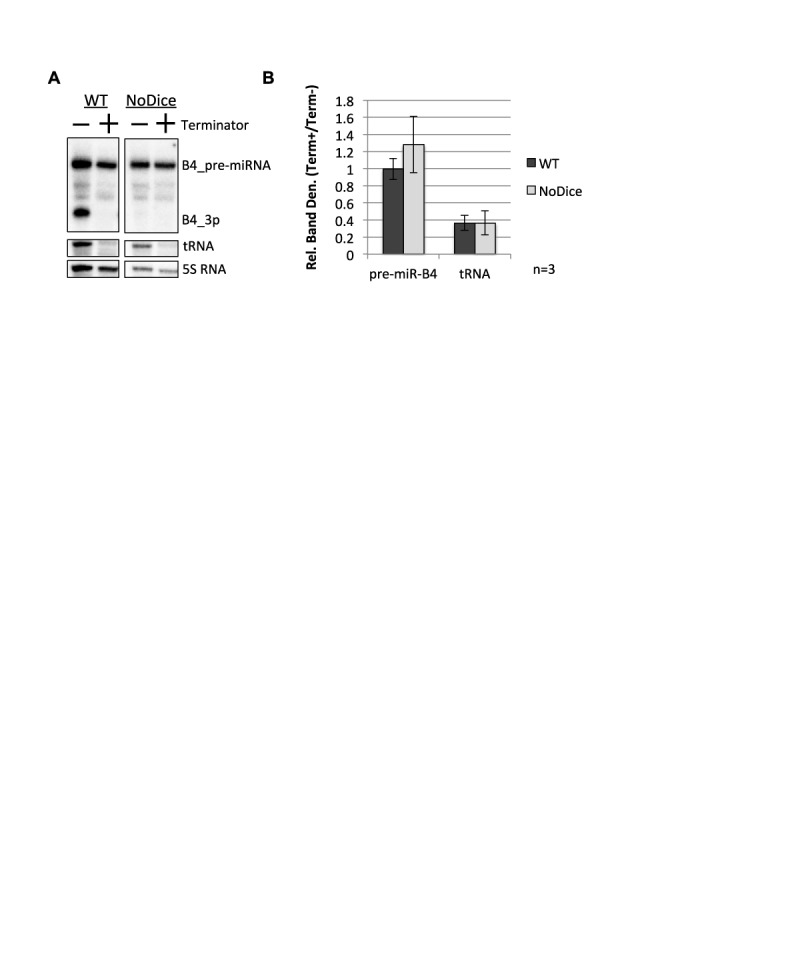
Conversion of the BLV pre-miRNA to a 5′-monophosphate does not precede Dicer processing. (**A**) 5′-end characterization of BLV-pre-miR-B4. Northern blot analysis was performed on small RNAs purified from either HEK293T or HEK293T NoDice cells transfected with the pBLV-B4 miRNA expression vector and treated with the indicated enzymes. (**B**) Graphical representation of the northern blot analysis band density ratio (Terminator+/Terminator−) of BLV-pre-miR-B4 and tRNA in either HEK293T cells (WT) and HEK293T NoDice cells (NoDice) relative to the 5S RNA. The bars represent the average ratio of three independent biological replicates +/− the standard deviation (*n* = 3).

The above data identify a tri-phosphatase activity involved in the biogenesis of BLV miRNAs. We wished to narrow in on the substrate of this phosphatase. Although a large fraction of the pre-miRNAs are 5′-triphosphorylated and readily processed by Dicer *in vitro* (Figure 4F; Supplementary Figure 2E), it remained possible in cells that the 5′-triphosphorylated pre-miRNAs were converted to a 5′-monophosphate before being rapidly cleaved by Dicer into the derivative miRNAs. Such a model predicts that in cells without Dicer activity, there should be an accumulation of 5′**-**monophosphorylated pre-miRNA. To test this, we transfected the pBLV-B4 expression vector into HEK293T (WT) and HEK293T Dicer knockout (NoDice) cells (32), isolated RNA and performed 5′-end characterization. As expected, tRNA from both WT and NoDice cells was readily degraded with Terminator^TM^, while the 5S RNA remained largely unchanged (Figure 6A and B). BLV-pre-miR-B4 from both the WT and NoDice cells remained largely unaffected by Terminator^TM^ treatment (Figure 6A and B). This indicates that the amount BLV-pre-miR-B4 that contains a 5′-monophosphate did not measurably increase in the NoDice cells (Figure 6A,B). These data demonstrate that the BLV pre-miRNAs are not readily converted to a 5′-monophosphate preceding Dicer processing, implying that any tri-phosphatase activity on the pre-miRNAs requires Dicer, or alternatively, that the tri-phosphatase activity occurs on the derivative miRNAs subsequent to Dicer processing.

**Figure 7.**
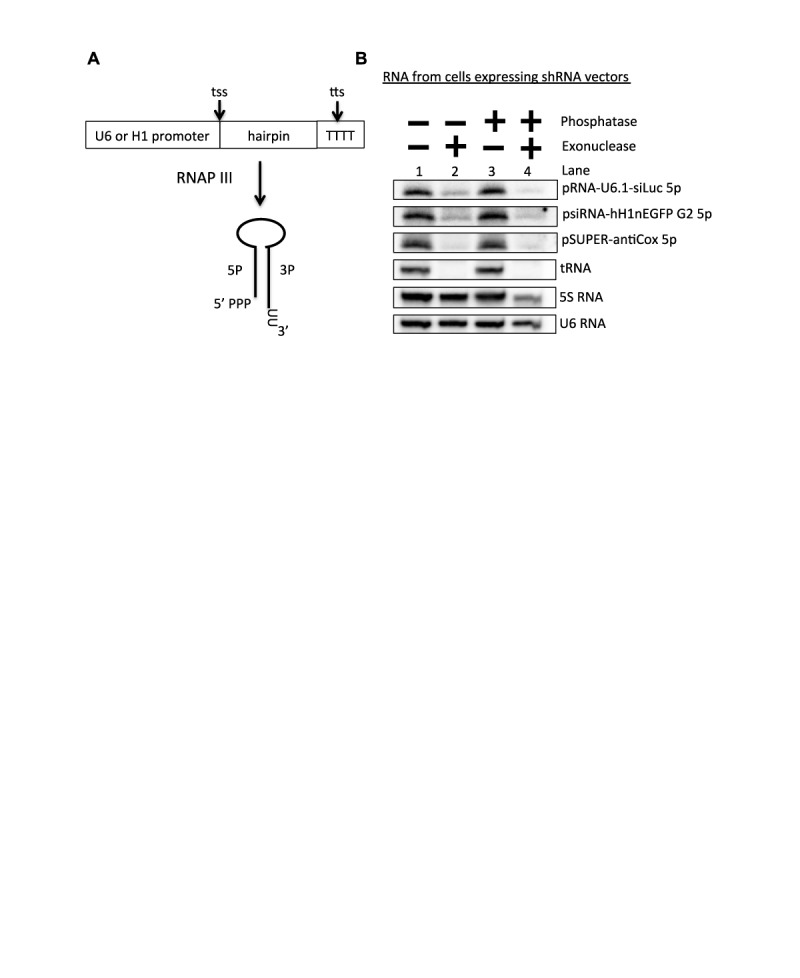
The 5′-end of 5p sRNAs derived from shRNAs contains a 5′-monophosphate. (**A**) Schematic diagram of shRNA biogenesis. (**B**) 5′-end characterization of 5p sRNAs derived from RNAP III-generated shRNAs. Northern blot analysis was performed on small RNAs isolated from HEK293T cells transfected with the indicated shRNA vectors (pRNA-U6.1-siLuc, psiRNA-hH1nEGFP G2, pSUPER-antiCox) and treated with the indicated enzymes.

### 5p sRNAs derived from RNAP III-generated shRNAs contain a 5′-monophosphate

RNAP III-generated shRNAs are generally designed so that the 5′ and 3′ ends of the pre-miRNA hairpin are the RNAP III transcription start site and the transcription termination site, respectively (Figure 7A). Thus, shRNA primary transcripts are intrinsically 5′-triphosphorylated. Our above data suggest that the BLV pre-miRNAs are generated in an analogous manner, yet the majority of the derivative BLV 5p miRNAs contain a 5′-monophosphate. Thus, we hypothesized that the 5p sRNAs derived from shRNAs will also contain a 5′-monophosphate. To test this, we performed 5′-end characterization on the 5p sRNAs generated from three different shRNAs that are driven by either the U6 promoter (pRNA-U6.1-siLuc) or the H1 promoter (psiRNA-hH1nEGFP G2 and pSUPER-antiCox1). Terminator^TM^ treatment readily decreased the 5p sRNAs derived from all three shRNAs without RNA 5′ polyphosphatase treatment, while the 5S RNA was unaffected (Figure 7B lanes 1 and 2). This is consistent with the 5p sRNAs containing a 5′-monophosphate. These results indicate that, similar to the BLV 5p miRNAs, shRNA-derived 5p sRNAs contain a 5′-monophosphate.

## DISCUSSION

**Figure 8.**
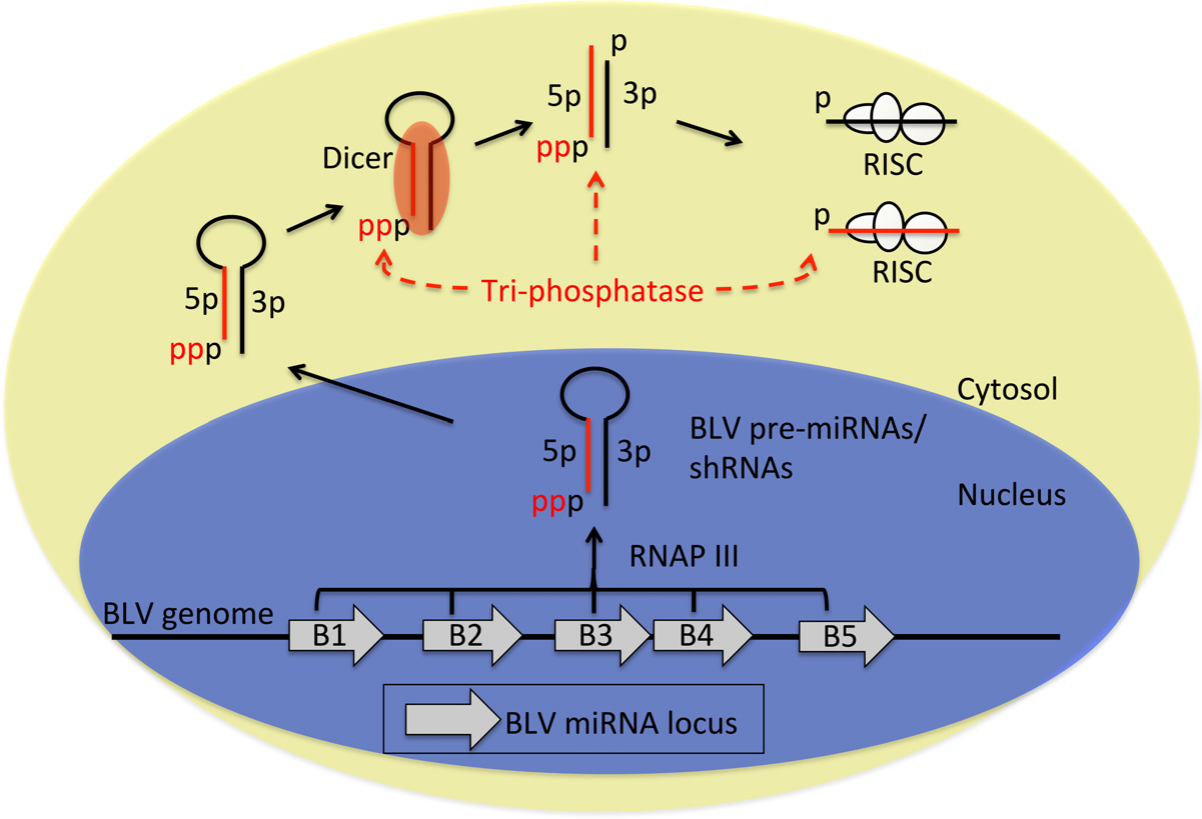
Model of the BLV biogenesis pathway. Schematic diagram modeling the BLV miRNA biogenesis pathway. Dashed red arrows indicate possible substrates for dephosphorylation by an RNA tri-phosphatase.

Recent studies have identified miRNAs encoded by foamy viruses, which belong to the *Spumaretrovirinae* subfamily, and BLV, which belongs to the *Orthoretrovirinae* subfamily (24,25,43,44). Both the foamy viruses and BLV express miRNAs via subgenomic RNAP III transcription, while the viral mRNAs are resistant to Drosha cleavage. This miRNA biogenesis route does not involve cleavage of the viral genomic RNA thereby avoiding a potential penalty in fitness. Here, we further analyze the biogenesis pathway of the BLV miRNAs and propose that an as yet-to-be-identified RNA 5′-tri-phosphatase(s) plays a role in the biogenesis pathway of the BLV miRNAs and RNAP III-generated shRNAs (Figure 8).

In agreement with our previous characterization of BLV-miR-B4 (24), our data demonstrate that all five BLV pri-miRNAs are expressed independent of RNAP II transcription (Figure 1A). Furthermore, each BLV pre-miRNA-encoding region independently expresses the corresponding pre- and mature-miRNAs (Figure 1C), suggesting that the BLV pre-miRNAs are individually transcribed and processed. Mutagenesis within the regions of BLV-miR-B1 and BLV-miR-B4 demonstrated that BLV pre-miRNA expression requires an A box(es) in the 5p arm/loop, a RNAP III transcription termination signal at the 3′ end of the hairpin, and a B-box sequence downstream of the hairpin (Figure 2). Combined, these data indicate that each BLV pre-miRNA is individually transcribed by RNAP III from an independent, compact RNAP III type II gene. This novel RNAP III promoter/gene architecture may be useful in designing more compact shRNA expression systems.

Previous work demonstrated that Drosha does not liberate the BLV pre-miRNA hairpins in the context of either RNAP II or RNAP III transcripts (24). However, the mechanism of pre-miRNA production had remained unclear. Our results show that RNAP III transcription termination generates the 3′-end of the pre-miRNA hairpins (Figures 2 and 3). The position of the A box elements (Figure 2) and the 5′-purine on many of the BLV pre-miRNAs and 5p miRNAs that corresponds to a canonical RNAP III transcription initiation site (Supplementary Table S2) suggests that RNAP III directly generates the 5′-end of each pre-miRNA hairpin. Indeed, our enzymatic characterization of the BLV pre-miRNAs revealed that a large fraction of the BLV pre-miRNAs are 5′-triphosphorylated (Figure 4C–F), characteristic of primary RNAP III transcripts. Thus, similar to manmade shRNAs, these results demonstrate that RNAP III directly transcribes the BLV pre-miRNA hairpins, whereby the transcription initiation site and the transcription termination site generate the 5′- and 3′-ends, respectively.

Though our data support that RNAP III directly generates the 5′-end of the majority of BLV pre-miRNA hairpins, longer RNAs, spanning the pre-miRNA region and containing a 5′ extended region (Supplementary Figure S2A), are observed upon transient transfection of some BLV miRNA expression vectors (24). Importantly, these RNAs are not detected during infection (24), are generally low in abundance relative to the respective pre- and mature-miRNAs, and are not observed for all the BLV miRNAs (Figures 1A, C and 3C). These observations suggest that these RNAs are likely aberrant transcripts resulting from promiscuous RNAP III initiation upstream of the 5′-end of the pre-miRNA during transient transfection. Nonetheless, formally our data cannot exclude the possibility that a small fraction of the BLV pre-miRNAs may be generated from these longer RNAs by an unprecedented mechanism.

Our data indicate that the majority of BLV pre-miRNAs are 5′-triphosphorylated, while the derivative 5p arms of BLV-miR-B2 and BLV-miR-B5 are primarily 5′-monophosphorylated (Figure 5B–D). RNA-seq analysis revealed that the 5′-nucleotide of the predominant 5′-triphosphorylated BLV-pre-miR-B5 isoform is identical to the 5′-nucleotide of the predominant BLV-miR-B5 5p isoform (Figure 5E). Similar phenomena were observed for the other BLV pre-miRNAs and derivative 5p miRNAs (Supplementary Figure S2). We note that for some pre-miRNA isoforms, low or no read counts were obtained (Supplementary Table S2). As these RNAs are readily detected via northern blot analysis, we suspect the secondary structure may inhibit inclusion into our small RNA library. Nevertheless, as the pre-miRNAs that we were able to sequence show identical 5′-nucleotides to the derivative 5p miRNAs, this supports that a tri-phosphatase activity, as opposed to a nuclease activity, results in the 5′-monophosphate on the BLV 5p miRNAs.

Whether the pre-miRNA and/or 5p miRNAs are subject to dephosphorylation remains unclear. However, our data demonstrate that Dicer is able to process the 5′-triphosphorylated BLV pre-miRNAs into miRNAs (Figure 4F; Supplementary Figure S2E and F), suggesting that the derivative 5′-triphosphorylated 5p miRNAs may be subject to dephosphorylation. Further supporting this, we did not observe an increase in 5′-monophoshphorylated BLV-pre-miR-B4 in NoDice cells (Figure 6), which indicates that the BLV pre-miRNAs are not readily dephosphorylated preceding Dicer association/processing. Combined, these data suggest that the putative tri-phosphatase dephosphorylates the BLV pre-miRNAs concurrent with Dicer processing or the derivative 5p miRNAs subsequent to Dicer processing (Figure 8).

The natural substrate(s) and natural function(s) of the phosphatase activity we have uncovered are unknown. Two non-mutually exclusive hypotheses for these include: (i) This phosphatase activity regulates the entry of, or is required for host RNAs to ‘enter into’ their functional machinery. (ii) As 5′-triphosphorylated RNAs are known to activate the innate immune response (45–47), this phosphatase activity may limit the immunostimulatory properties of some host RNP III-transcribed RNAs. Thus, we speculate that the BLV pre- and/or mature- miRNAs may take advantage of this host tri-phosphatase activity to remove the 5′-triphosphate in order to allow access to the miRNA silencing machinery, and/or alternatively to dampen or prevent the host immune response. Determining the enzyme(s) responsible for this tri-phosphatase activity is an important issue not only for understanding retroviral miRNA biogenesis, but also to better predict the efficacy of RNAP III-generated shRNAs (and associated silencing-competent star strands) in different tissues and physiological conditions.

In summary, we have characterized the BLV miRNA biogenesis pathway, demonstrating that each BLV pre-miRNA is directly transcribed by RNAP III from a novel, compact RNAP III type II gene. We identify a previously unknown tri-phosphatase activity involved in the biogenesis of BLV miRNAs and shRNAs. These findings further our understanding of retroviral miRNA biogenesis and have implications for the design and optimal utilization of shRNAs.

## SUPPLEMENTARY DATA

Supplementary Data are available at NAR Online.

SUPPLEMENTARY DATA
