# Supplementary material for: Identification of tri-phosphatase activity in the biogenesis of retroviral microRNAs and RNAP III-generated shRNAs
Source: Nucleic Acids Res. 2014 Nov 26;42(22):13949–62. doi: 10.1093/nar/gku1247 (PMC4267658; doi:10.1093/nar/gku1247)
Supplement: SUPPLEMENTARY DATA [file supp_42_22_13949__index.html]

Identification of tri-phosphatase activity in the biogenesis of retroviral microRNAs and RNAP III-generated shRNAs — Identification of tri-phosphatase activity in the biogenesis of retroviral microRNAs and RNAP III-generated shRNAs — SUPPLEMENTARY DATA 

# Identification of tri-phosphatase activity in the biogenesis of retroviral microRNAs and RNAP III-generated shRNAs

## SUPPLEMENTARY DATA

**Files in this Data Supplement:**

- SUPPLEMENTARY DATA
