# Supplementary material for: Identification of tri-phosphatase activity in the biogenesis of retroviral microRNAs and RNAP III-generated shRNAs
Source: Nucleic Acids Res. 2014 Nov 26;42(22):13949–62. doi: 10.1093/nar/gku1247 (PMC4267658; doi:10.1093/nar/gku1247)
Supplement: SUPPLEMENTARY DATA [file supp_gku1247_nar-02352-y-2014-File010.pdf]

Supplementary Figure S1

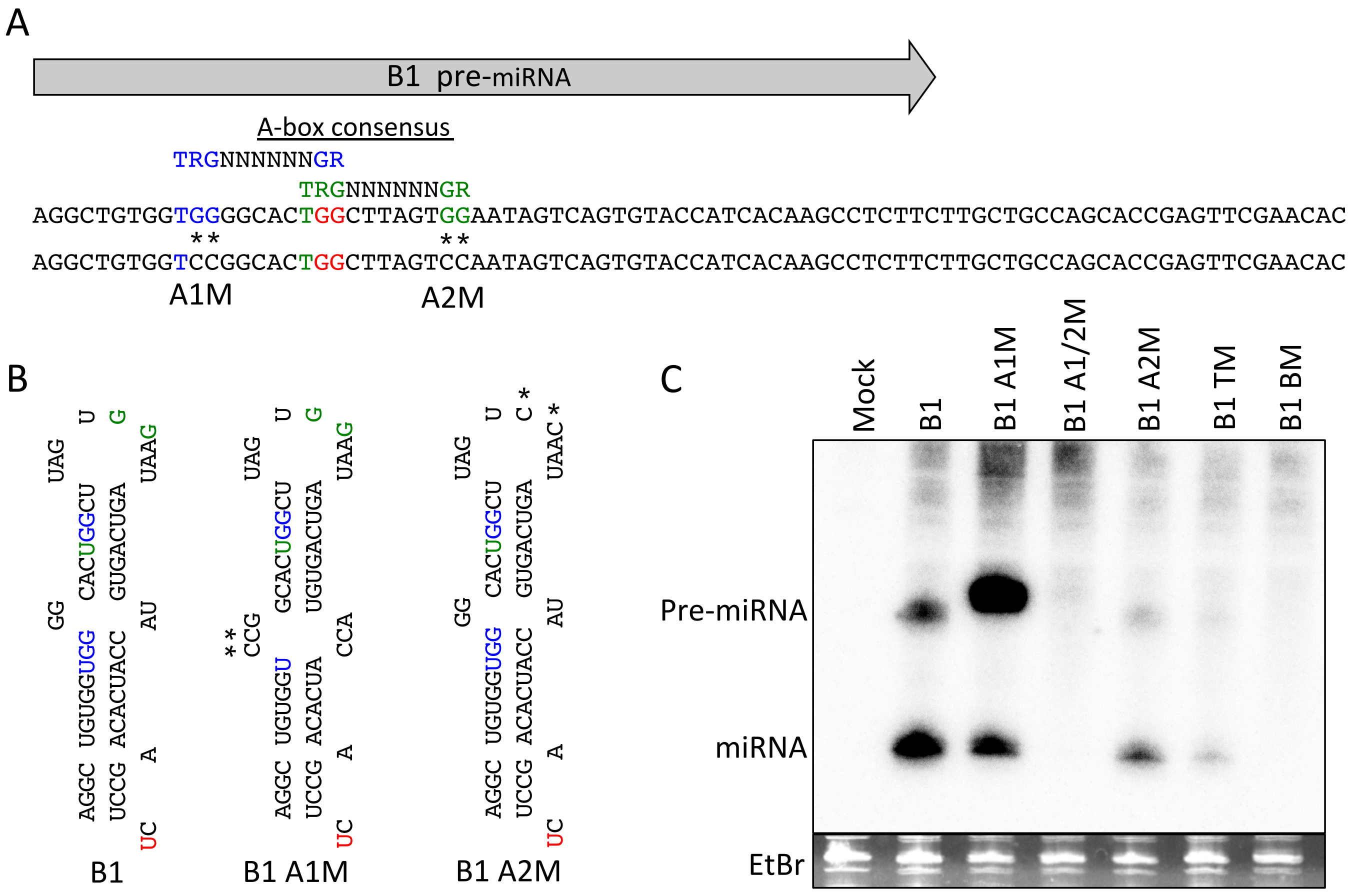

Supplementary Figure S1. Analysis of the BLV-miR-B1 RNAP III-transcriptional elements.
(A) Schematic diagram of the overlapping A-box sequences in the BLV-miR-B1 region. The consensus sequence specific for the upstream A-box (A-box-1) is indicated by blue letters, while the consensus sequence for the downstream A-box (A-box-2) is indicated by green letters. The overlapping consensus sequence is indicated by red letters. The (*) indicate point mutations in either A-box-1 (A1M) or A-box-2 (A2M) where indicated. (B) Predicted secondary structures of B1, B1 A1M, and B1 A2M. The (*) indicate mutations relative to B1. (C) Northern blot analysis of HEK293T cells transfected with pBLV B1 and indicated variants.

Supplementary Figure S2

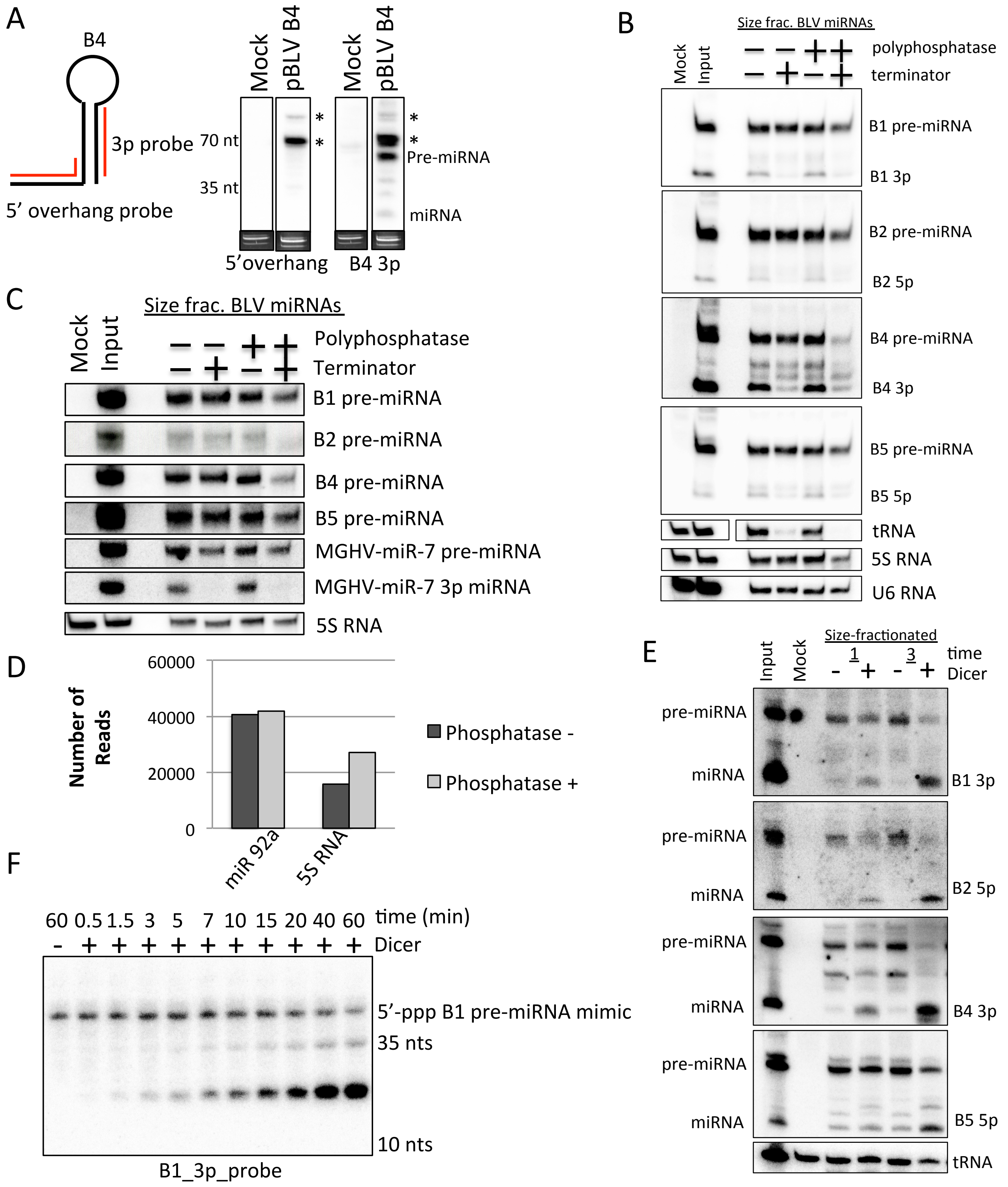

Supplementary Figure S2. BLV pre-miRNAs are RNAP III primary transcripts

(A) Northern blot analysis of RNA purified from HEK293T cells transfected with pBLV-B4. The blot was probed with a probe complementary the 3p miRNA arm and the 5´-flanking sequence. The asterisks indicate putative primary RNAP III transcripts longer than the BLV-pre-miR-B4. (B) Wider cropped Northern blot analysis of 5’-end characterization of the BLV pre-miRNAs and miRNAs shown in Figures (4B and 5C). (C) 5’-end characterization of the BLV pre-miRNAs and the MGHV-miR-M1-7 pre- and mature- miRNA. (D) RNA seq reads of miR-92a and the 5S RNA with or without RNA 5’ polyphosphatase treatment. (E) BLV pre-miRNAs were gel-purified from HEK293T cells transfected with pBLV miRNA expression vectors and incubated with (+) or without (-) Dicer for 1 or 3 hours. Northern blot analysis was performed on the the RNA and probed with the indicated probes. (F) In vitro Dicer processing of the T7-transcribed, 5´-triphsophorylated BLV-pre-miR-B1 mimic. Reactions were stopped at the indicated time points.

Supplementary Table S1. Oligonucleotides used for cloning and Northern blot analysis

Oligo name	Sequence
SV40_3p	GGCATGAAACAGGCA
MGHV -M1-7–3p probe	AATAAAGGTGGGCGCGATATC
BLV_B1_3p_probe	AGAGGCTTGTGATGGTACACTGA
BLV_B2_5p_probe	TCTCTGCGCTACACTCAGTCAT
BLV_B3_3p_probe	AGAAATCGCCCCCGTCAGCGTTA
BLV_B4_3p_probe	AAAGGCGCAGAGACCGTGGTGCTA
FLK_B4_3p_probe	AAAGGCGCAGAGACTATGGTGCTA
B4 _3p_probe_(s)	AAGGCGCAGAGACTGTGGTGCTA
BLV_B5_5p_probe	ACCTCTGAGCCACAACCTTCCT
FLK_B5_5p_probe	ACCTCTGAGCCACAACCCTCCT
BLV_B4_5'-overhang_probe	CTCTCGCTTAGCACAGCTGC
BLV_B1_s	AGTCTCGAGCCTTCGACCCTGCCCTTGACACCCCCGTGTTTCACGCACCCTCAGGCTGTGGTGGGGCACTGGCTTAGTGGAATA
BLV_B1_a	TACTCTAGAGCTGTGTTCGAACTCGGTGCTGGCAGCAAGAAGAGGCTTGTGATGGTACACTGACTATTCCACTAAGCCAGTGCC
BLV_B2_s	gtacCTCGAGACAGCCCTACCCTGAGCCTCTCTGAGTACATGACTGAGTGTAGCGCAGAGAGGTTGTCGCTTCTGCGTGT
BLV_B2_a	tgcaTCTAGAAGGCAATGGGAGGGCGCGAACCCCAATCGGCTATAAAAAATGACTGAGTGACACGCAGAAGCGACAACCT
BLV_B3_s	TGACACTCGAGACGGTTAAGACCTCTCTCACTTCTGCTTCACCATCCCCCTGCCAGCGTTGGTCTAGTGGAAAGAACTAA
BLV_B3_a	GAACTCTAGACACCAGAGCCTCTCGCTTAGCACAGCTGCAAGAAATCGCCCCCGTCAGCGTTAGTTCTTTCCACTAGACC
BLV_B4_s	TGACACTCGAGCTGCAGCTGTGCTAAGCGAGAGGCTCTGGTGCTGGGGATAAGATGCGGCCCCTAGCACCA
BLV_B4_a	GAACTCTAGAGGCATGGGGAAGATTCGAACCCAAAAGGCGCAGAGACTGTGGTGCTAGGGGCCGCATCTT
BLV_B5_s	CAGTCTCGAGCTGAAAATCTCAGCTCGCACCCCAAGGAAGGTTGTGGCTCAGAGGTTAAAATAGCTCGGACCGCAACCTC
BLV_B5_a	GTACTCTAGATTAGGGGGCCCAGAACCCGGGGCCTTGCGAGGGTGGAATAAAAAGAAAGGGAGGTTGCGGTCCGAGCTAT
B13p_RR_ s	AGTCTCGAGAGAGGCTTGTGATGGTACACTGAATCGGCTAAGAGGC
B13p_RR_ a	CATTCTAGATCAGTGTACCATCACAAGCCTCTTAGCCGATTCAGTG
B25p_RR_ s	AGTCTCGAGTCTCTGCGCTACACTCAGTCATATCGGCTATCTCTG
B25p_RR_ a	CATTCTAGAATGACTGAGTGTAGCGCAGAGATAGCCGATATGACT
FLK_B23p_RR_s	AGTCTCGAGAAAATGACTGAGCGACACGCAGAATCGGCTAAAAATGACT
FLK_B23p_RR_a	CATGGGCCCTCTAGATCTGCGTGTCGCTCAGTCATTTTTAGCCGATTCT
B35p_ RR_s	AGTCTCGAGGACCAACGCTGGCAGGGGGATATCGGCTAGACCAA
B35p_ RR_a	CATTCTAGAATCCCCCTGCCAGCGTTGGTCTAGCCGATATCCCC
B33p_ RR_s	AGTCTCGAGAGAAATCGCCCCCGTCAGCGTTAATCGGCTAAGAAAT
B33p_ RR_a	CATTCTAGATAACGCTGACGGGGGCGATTTCTTAGCCGATTAACGC
B43p_RR_s	AGTCTCGAGGGCGCAGAGACTGTGGTGCTAGGGATCGGCTAGG
B43p_RR_s	CATTCTAGAccctagcaccacagtctctgcgccTAGCCGATccc
B55p_RR_s	AGTCTCGAGACCTCTGAGCCACAACCTTCCTATCGGCTAACCTCT
B55p_RR_a	CATTCTAGAAGGAAGGTTGTGGCTCAGAGGTTAGCCGATAGGAAG
B53p_RR_s	AGTCTCGAGAAAGAAAGGGAGGTTGCGGTCCGAGCATCGGCTAAAA
B53p_RR_a	CATTCTAGAGCTCGGACCGCAACCTCCCTTTCTTTTAGCCGATGCT
B4_ AM2_s	GCTGTGCTAAGCGAGAGGCTCTGGTGCTGGGGATActcTGCGGCCCCTAGCACCA
B4_ AM2_a	TGGGGAAGATTCGAACCCAAAAGGCGCAGAGACTGTGGTGCTAGGGGCCGCAgag
B4_AM1_s	CTGTGCTAAGCGAGAGGCTCTGGTGCTGGccATAAGATGCGGCggCTAGCACCAC
B4_AM1_a	ATGGGGAAGATTCGAACCCAAAAGGCGCAGAGACTGTGGTGCTAGccGCCGCATC
B4_BM_s	GCTGTGCTAAGCGAGAGGCTCTGGTGCTGGGGATAAGATGCGGCCCCTAGCACCA
B4_BM_a	TGGGGAAGATTCGttgCCAAAAGGCGCAGAGACTGTGGTGCTAGGGGCCGCATCT
B4_TM_s	CAGTCTCGAGCTCGAGCTGCAGCTGTGCTAAGCGAGAGGCTCTGGTGCTGGGGATAAGATGCGGCCCCTAGCACCAC
B4_TM_a	GTACTCTAGAGCTCTAGAGGCATGGGGAAGATTCGAACCCCCAAGGCGCAGAGACTGTGGTGCTAGGGGCCGCATCT
universal_B4_outer primer_s	TAAGCTCGAGCTGCAGCTGTGCTAAGCGAG
universal_B4_outer_primer_a	TTGATCTAGAGGCATGGGGAAGATTCG
univeral_B1_ s	AGTCTCGAGCCTTCGACCCTGCCCTTGACACCCCCGTGTTTCACGCACCCTCAGGCTG
B1_BM_TM_ s	CACGCACCCTCAGGCTGTGGTGGGGCACTGGCTTAGTGGAATAG
B1_BM_a	TACTCTAGAGGCTGTGTTCGTTGTCGGTGCTGGCAGCAAGAAGAGGCTTGTGATGGTACACTGACTATTCCACTAAGCCAGTGCC
B1_TM_a	TACTCTAGAGGCTGTGTTCGAACTCGGTGCTGGCAGCTTGTAGAGGCTTGTGATGGTACACTGACTATTCCACTAAGCCAGTGCC
B1_A1M_s	TTTCACGCACCCTCAGGCTGTGGTCCGGCACTGGCTTAGTGGAATAG
B1_A1M_a	TACTCTAGAGGCTGTGTTCGAACTCGGTGCTGGCAGCAAGAAGAGGCTTGTGATGGTACACTGACTATTCCACTAAGCCAGTGCC
B1_A1/2M_s	TTTCACGCACCCTCAGGCTGTGGTGGGGCACTCCCTTAGTGGAATAG
B1_A1/2M_ a	TACTCTAGAGGCTGTGTTCGAACTCGGTGCTGGCAGCAAGAAGAGGCTTGTGATGGTACACTGACTATTCCACTAAGGGAGTGCC
B1_A2M_s	TTTCACGCACCCTCAGGCTGTGGTGGGGCACTGGCTTAGTCCAATAG
B1_A2M_a	TACTCTAGAGGCTGTGTTCGAACTCGGTGCTGGCAGCAAGAAGAGGCTTGTGATGGTACACTGACTATTGGACTAAGCCAGTGCC
BLV913_3'UTR_TM_5'flank_s_ApaL1	GGTGGTGCACTGGCTTAGTGGAAT
	CTTAGTGGAATAGTCAGTGTACCATCACAAGCCTCTGCCAGCTGCCAGCACCGAGTTCGAACACAGCCCTACCCTGAGCCTCTCTGAGTGCATGAC<br>TGAGTGTAGCGCAGAGAGATTGTCGCTTCTGCGTGTCGCTCAGTCAGACCAAATAGCCGATTGGGGTTCGCGCCCTTCCGTTGCCTGTGACACAG<br>ATAAGACCTCTCTCACTTCTGCTTCACCATCCCCCTGCCAGCGTTGGTCTAGTGGAAAGAACTAACGCTGACGGGGGCGATGCAAGCAGCTGTGCT<br>AGCGGGAGGCTCTGGTGCTGGGGATAAGATATGGCCCTTAGCACCATAGTCTCTGCGCCTACGGGGTTCAAATCTTCCCCACGCAGCTTCCGCTTT<br>TTACGCCCTGTTGCACACCCTTTCTAGAGATACCTGAAAATCTCAGCTCGCACCCTGAGGAGGGTTGTGGCTCAGAGGTTAAAATAGCTCGAGCCG
BLV913_miRNAs_TM_gblock	CAACCTCCCTTCCAGGACATT
BLV UTR 90 bp 3' frag as	TGGACCCGAGCATCCCCTAGTAGAGGAAATTTTGAACCTCCGTTAGGGGGCTCAGAACCCGGGGCCTTGCGAGGGTGGAATGTCCTGGAA

Supplementary Table S1 cont.

Oligo name	sequence
BLV913_3'UTR_TM_3'flank_a_ApaL1	ATTGGTGCACACTTGGACCCGAGCATCC
BLV913_ 3'UTR_ s_sal1	TAGAGTCGACGTCTCACTCTCACTCTCCTC
BLV913_ 3'UTR_ a_spe1	ATAGACTAGTGCAAGCCAGACGCCCTT
pRNA-U6.1-siLuc_5p_probe	TCGAAGTACTCAGCGTAAG
psiRNA-hH1nEGFP-G2_5p_probe	TGAACTTCAGGGTCAGCTTGC
pSUPERantiCox1_5p_probe	AATCCTCTGGAGTGTTCTT
U6_probe	CGTTCCAATTTTAGTATATGTGCTGCC
5S_probe	CCCTGCTTAGCTTCCGAGATCAGAC
GAPDH_sense primer	GTCAGTGGTGGACCTGACCT
GAPDH_antisense primer	CAGTTGCCATGTAGACCCCTT
T7-B1_pre-miRNA mimic sen	TAATACGACTCACTATAGGGCTGTGGTGGGGCACTGGCTTAGTGGAATAGTCAGTGTACCATCACAAGCCTCT
T7-B1_pre-miRNA mimic antisense	AGAGGCTTGTGATGGTACACTGACTATTCCACTAAGCCAGTGCCCCACCACAGCCCTATAGTGAGTCGTATTA

Supplementary Table S2. RNA-seq reads mapped to the BLV miRNA loci from RNA pre-treated with (P+) or without (P-) RNA 5'-polyphosphatase

	Sequence	Read count (P-)	Read count (P +)	Normalized read count (P+)
B1 reference sequence	CTCAGGCTGTGGTGGGGCACTGGCTTAGTGGAATAGTCAGTGTACCATCACAAGCCTCTTCTTGCT			
B1 pre-miRNA-sized RNAs	GCTGTGGTGGGGCACTGGCTTAGTGGAATAGTCAGTGTACCATCACAAGCCTCTT	7	108	122
	GCTGTGGTGGGGCACTGGCTTAGTGGAATAGTCAGTGTACCATCACAAGCCTCTTCTT		27	30
	GCTGTGGTGGGGCACTGGCTTAGTGGAATAGTCAGTGTACCATCACAAGCCTCTTCT		21	24
	GCTGTGGTGGGGCACTGGCTTAGTGGAATAGTCAGTGTACCATCACAAGCCTC		14	16
	GCTGTGGTGGGGCACTGGCTTAGTGGAATAGTCAGTGTACCATCACAAGCCTCT		7	8
	AGGCTGTGGTGGGGCACTGGCTTAGTGGAATAGTCAGTGTACCATCACAAGCCTCT	1	1	1
	GGCTGTGGTGGGGCACTGGCTTAGTGGAATAGTCAGTGTACCATCACAAGCCTCT		1	1
	AGGCTGTGGTGGGGCACTGGCTTAGTGGAATAGTCAGTGTACCATCACAAGCCTCTT	3		
	GCTGTGGTGGGGCACTGGCTTAGTGGAATAGTCAGTGTACCATCACAAGCCTCTTCTT	3		
B1_5p	AGGCTGTGGTGGGGCACTGGCT	573	624	704
	AGGCTGTGGTGGGGCACTGGC	190	297	335
	GCTGTGGTGGGGCACTGGCT	6	17	19
	GCTGTGGTGGGGCACTGGC	4	28	32
B1_3p	TCAGTGTACCATCACAAGCCTCT	6843	6067	6843
B2 refernce sequence	GAGTACATGACTGAGTGTAGCGCAGAGAGGTTGTCGCTTCTGCGTGTCACTCAGTCATTTTTTATA			
B2 pre-miRNA-sized RNAs	AGTACATGACTGAGTGTAGCGCAGAGAGGTTGTCGCTTCTGCGTGTCACTCAGTCATT		8	11
	ACATGACTGAGTGTAGCGCAGAGAGGTTGTCGCTTCTGCGTGTCACTCAGTCATTT	5	6	9
	ACATGACTGAGTGTAGCGCAGAGAGGTTGTCGCTTCTGCGTGTCACTCAGTCATTTT		6	9
	ACATGACTGAGTGTAGCGCAGAGAGGTTGTCGCTTCTGCGTGTCACTCAGTCAT		2	3
B2 _5p	ACATGACTGAGTGTAGCGCAGA	6014	3606	5172
	ATGACTGAGTGTAGCGCAGA	737	431	618
	ACATGACTGAGTGTAGCGCAG	531	468	671
B2 3p	TGCGTGTCACTCAGTCATTTT	1202	838	1202
	TGCGTGTCACTCAGTCATTTTT	190	146	209
B4 refernce sequence	CTAAGCGAGAGGCTCTGGTGCTGGGGATAAGATGCGGCCCCTAGCACCACAGTCTCTGCGCCTTTTGGGTTC			
B4 pre-miRNA-sized RNAs	GAGAGGCTCTGGTGCTGGGGATAAGATGCGGCCCCTAGCACCACAGTCTCTGCGCCTTT	79	791	445
	GAGAGGCTCTGGTGCTGGGGATAAGATGCGGCCCCTAGCACCACAGTCTCTGCGCCTTTT	36	297	167
	GAGAGGCTCTGGTGCTGGGGATAAGATGCGGCCCCTAGCACCACAGTCTCTGCGCCTT	5	50	
	GCGAGAGGCTCTGGTGCTGGGGATAAGATGCGGCCCCTAGCACCACAGTCTCTGCGCCTTT	3		
	GAGGCTCTGGTGCTGGGGATAAGATGCGGCCCCTAGCACCACAGTCTCTGCGCCTTT	3		
	AGGCTCTGGTGCTGGGGATAAGATGCGGCCCCTAGCACCACAGTCTCTGCGCCTTT	3		
	GAGAGGCTCTGGTGCTGGGGATAAGATGCGGCCCCTAGCACCACAGTCTCTGCGCCT	2	8	5
	AGAGGCTCTGGTGCTGGGGATAAGATGCGGCCCCTAGCACCACAGTCTCTGCGCCTTT	1	15	8
	AGAGGCTCTGGTGCTGGGGATAAGATGCGGCCCCTAGCACCACAGTCTCTGCGCCTTTT	1		0
B4_5p	AAGCGAGAGGCTCTGGTGCTGG	89	66	37
	AGCGAGAGGCTCTGGTGCTGG	65	69	39
	AAGCGAGAGGCTCTGGTGCTGGG	40	59	33
	AGCGAGAGGCTCTGGTGCTG	36		
	AAGCGAGAGGCTCTGGTGCT	24		
	GAGAGGCTCTGGTGCTGGGGA	10	40	23
	GAGAGGCTCTGGTGCTGGGG	8	40	23
	GAGAGGCTCTGGTGCTGGG	7	17	10
	GAGAGGCTCTGGTGCTGGGGAT	5		
	AGCGAGAGGCTCTGGTGCTGGG		77	43
B4_3p	TAGCACCACAGTCTCTGCGCCTTT	3132	5566	3132
	TAGCACCACAGTCTCTGCGCCTT	1596	2939	1654
	TAGCACCACAGTCTCTGCGCCTTTT	389	645	363
	TAGCACCACAGTCTCTGCGCCT	253	475	267
B5 (NC_001414) reference sequence	TCTCAGCTCGCACCCCAAGGAAGGTTGTGGCTCAGAGGTTAAAATAGCTCGGACCGCAACCTCCCTTTCTTTTT			
B5 pre-miRNA-sized RNAs	AAGGAAGGTTGTGGCTCAGAGGTTAAAATAGCTCGGACCGCAACCTCCCTT	170	502	758
	CAAGGAAGGTTGTGGCTCAGAGGTTAAAATAGCTCGGACCGCAACCTCCCT	46	59	89
	CCAAGGAAGGTTGTGGCTCAGAGGTTAAAATAGCTCGGACCGCAACCTCCC	5	8	12
B5_5p	AAGGAAGGTTGTGGCTCAGAGGT	2398142	1240202	1872688
	AAGGAAGGTTGTGGCTCAGAGG	472546	334894	505685
	AAGGAAGGTTGTGGCTCAGAG	154662	101656	153499
	AAGGAAGGTTGTGGCTCAGA	141431	85484	129080
	CAAGGAAGGTTGTGGCTCAGAGGT	11655	6795	10260
	CCAAGGAAGGTTGTGGCTCAGA	1349	927	1400
	CCAAGGAAGGTTGTGGCTCAGAG	928	650	981
B5_3p	CTCGGACCGCAACCTCCCTTTC	118851	78710	118851
	CTCGGACCGCAACCTCCCTTTCT	68792	31244	47178
	CTCGGACCGCAACCTCCCTTT	37693	18822	28421
	CTCGGACCGCAACCTCCCTT	3379	1592	2404

Supplementary Table S2. cont.

	Sequence	Read count (P-)	Read count (P +)	Normalized read count (P+)
B5 (913) refernce sequence	GCACCCTGAGGAGGGTTGTGGCTCAGAGGTTAAAATAGCTCGGACCGCAACCTCCCTTTCTTTTTATT			
B5_pre-miRNA	ACCCTGAGGAGGGTTGTGGCTCAGAGGTTAAAATAGCTCGAGCCGCAACCTCCCTT		5	5
	ACCCTGAGGAGGGTTGTGGCTCAGAGGTTAAAATAGCTCGAGCCGCAACCTCCCTTTC		3	3
	GAGGGTTGTGGCTCAGAGGTTAAAATAGCTCGAGCCGCAACCTCCCTTTCTTT		2	2
	GAGGGTTGTGGCTCAGAGGTTAAAATAGCTCGAGCCGCAACCTCCCTTTCTTTT		2	2
	GAGGAGGGTTGTGGCTCAGAGGTTAAAATAGCTCGAGCCGCAACCTCCCTTT	1	2	2
	AGGAGGGTTGTGGCTCAGAGGTTAAAATAGCTCGAGCCGCAACCTCCCTTTCT		1	1
	AGGGTTGTGGCTCAGAGGTTAAAATAGCTCGAGCCGCAACCTCCCTTT		1	1
	GAGGGTTGTGGCTCAGAGGTTAAAATAGCTCGAGCCGCAACCTCCCTTT		1	1
	CTGAGGAGGGTTGTGGCTCAGAGGTTAAAATAGCTCGAGCCGCAACCTCCCTTTCT	1		
	AGGAGGGTTGTGGCTCAGAGGTTAAAATAGCTCGAGCCGCAACCTCCCTTTCTTT	1		
B5_5p	GAGGAGGGTTGTGGCTCAGAGGT	866	781	823
	GAGGAGGGTTGTGGCTCAGAGG	166	224	236
	GAGGAGGGTTGTGGCTCAGAGGTT	109	165	174
	AGGAGGGTTGTGGCTCAGAGGT	126	128	135
B5_3p	CTCGAGCCGCAACCTCCCTTTCT	157	149	157

Table S2. Small RNA sequencing read counts for BLV pre-miRNA-sized RNAs and abundant miRNA isoforms that map to the respective BLV miRNA genomic locus with (P+) or without (P-) RNA 5' polyphosphatase treatment. 5p read and pre-miRNA read counts were normalized to their respective dominant 3p miRNA isoform.
